# Lysogenic *Streptococcus suis* Isolate SS2-4 Containing Prophage SMP Showed Increased Mortality in Zebra Fish Compared to the Wild-Type Isolate

**DOI:** 10.1371/journal.pone.0054227

**Published:** 2013-01-10

**Authors:** Fang Tang, Wei Zhang, Chengping Lu

**Affiliations:** Key Lab Animal Bacteriology, Ministry of Agriculture, College of Veterinary Medicine, Nanjing Agricultural University, Nanjing, China; University of York, United Kingdom

## Abstract

*Streptococcus suis* (*S. suis*) infection is considered to be a major problem in the swine industry worldwide. Based on the capsular type, 33 serotypes of *S. suis* have been described, with serotype 2 (SS2) being the most frequently isolated from diseased piglets. Little is known, however, about the pathogenesis and virulence factors of *S. suis*. Research on bacteriophages highlights a new area in *S. suis* research. A *S. suis* serotype 2 bacteriophage, designated SMP, has been previously isolated in our laboratory. Here, we selected a lysogenic isolate in which the SMP phage was integrated into the chromosome of strain SS2-4. Compared to the wild-type isolate, the lysogenic strain showed increased mortality in zebra fish. Moreover the sensitivity of the lysogenic strain to lysozyme was seven times higher than that of the wild-type.

## Introduction

*Streptococcus suis* serotype 2 (SS2) is a Gram-positive pathogenic bacterium capable of infecting piglets and can cause serious diseases such as arthritis, meningitis, and septicemia [1]. *S. suis* is also an important zoonotic agent that mainly afflicts humans in close contact with contaminated pigs or pig meat, causing meningitis and endocarditis [2], [3]. In 1998, 25 humans were shown to be infected with *S. suis* serotype 2 in the Jiangsu Province in China. Fourteen of these individuals died as a result [4]. In July 2005, a widespread outbreak of *S. suis* infection in humans emerged in the Sichuan province in China, involving 215 patients. This outbreak was accompanied by several sporadic cases of *S. suis* infections in humans in other provinces [5], [6], [7].

So far, little is known about the virulence and control of *S. suis* infections. Several prophages have been identified in published *S. suis* genomes [5], [6], [7]. In addition, we previously identified and sequenced a bacteriophage, designated SMP, capable of causing lytic infection in *S. suis* SS2-4. SMP, which was isolated in 2005 from nasal swabs obtained from healthy Bama minipig (Guizhou line), is the only *S. suis* phage that has been detected as in the form of active virions to date. [8]. Ten of 53 *S. suis* isolates investigated could be lysed by SMP. SMP had an isometric head of 50 nm, a non-contractile tail of approximately 135 nm [8], and a circular double-stranded DNA genome of 36,019 base pairs (bp).

Numerous bacterial virulence factors (VFs) have been shown to be encoded by prophages [9]. Human diseases directly caused by prophage encoded VFs include (but are not limited to) botulism, diphtheria, cholera, and those associated with Shiga toxigenic *Escherichia coli* such as *E. coli* O157 [10]. Other pathogenic species of *Streptococcus*, such as *S. pneumoniae* [11], group A Streptococcus [12], *S. pyogenes* [13] and *S. equi* [14] harbor prophages, some of which can be associated with increased virulence. Here we report the selection of a lysogenic *S. suis* serotype 2 isolate (SS2-4) containing the phage SMP integrated into its genome. The possible role of the prophage in colonization and pathogenesis of the lysogenic isolate was studied.

## Materials and Methods

### 1.1. Bacterial strain, phage and growth conditions

The *S. suis* serotype 2 strain SS2-4 and its lytic bacteriophage SMP were used in this study. The strain SS2-4 was isolated in Jiangsu in 1999 and stored in our laboratory. SS2-4 was cultured overnight at 37°C with 5% CO_2_ in Todd-Hewitt broth ([THB], BD, USA, Cat. No. 249240) or on Luria-Bertani (LB) agar supplemented with 6% sheep blood. The SMP was isolated as a free phage from nasal swabs obtained from healthy Bama minipig (Guizhou line) in 2005 [8].

#### 1.1.1 plaque assay

For phage infection bacterial cultures were supplemented with 10 mM CaCl_2_. Sandwich plaque assays were performed as described [15] with modifications. Briefly, overnight cultures in THB were diluted 1:100 in fresh THB and incubated for 8 h. Phage SMP lysate (200 μl, 10^6^ plaque forming units per ml [PFU/ml]) was added to bacterial culture and the culture was further incubated for 15 min at 37°C. The mixed culture was then poured into molten THB (5 ml) containing 0.5% agar and spread onto THB plates. The plates were incubated at 37°C for 12 h for plaque formation.

### 1.2. Selection of lysogenic SS2-4

Overnight cultures of SS2-4 (200 μl) were spread onto a THB plate. Spot assays were performed by spotting 10 μl of a phage SMP lysate (10^6^ PFU/ml) onto a previously seeded plate. The spot was allowed to dry and the plate was incubated overnight at 37°C. Surviving colonies were isolated from within a zone of lysis and incubated in THB medium at 37°C overnight. Putative lysogenic colonies were subcultured five times to remove unstable lysogeny(bacteria cells with a high rate of spontaneous induction). The rate of spontaneous induction of the lysogenic strain was tested as previously described [16].

#### 1.2.1. PCR conditions

To verify the presence of prophage SMP in the putative lysogenic strain, PCR assays were performed to amplify various SMP genes (Table 1). All PCR reactions were performed in a 25 μl reaction volume, containing 12.5 μl of Premix Taq (TaKaRa, Japan, code. D334), 8.5 μl of distilled water, 2 μl of the target DNA solution, 1 μl of forward primer (10 μM) and 1 μl of reverse primer (10 μM). The following amplification conditions were used: 5 min at 95°C for initial denaturing; 30 cycles of 1 min at 95°C, 30 sec at 56°C and 1 min at 72°C; 10 min at 72°C for extension.

**Table 1:** Primer sequences used in this study.

Primer	Sequence (5′-3′)	Gene	Amplicon size (bp)
P1U	ATCTGCATGGCCCACACGGTC	SMP integrase	823
P1L	CAAACGGACCTCTATCGCCTC		
P2U	TCGTCGTCTTCGGTTAGG	SMP repressor	647
P2L	TTGAGGTTTGGGTCTGTC		
P3U	TAGCGGTGCTGGTGGTCATAC	SMP lyase	817
P3L	TCCACCCTCGCAAGCTGGTCT		
P4U	TGCAGGCAAAGTACGTTTCCA	SMP major tail protein	248
P4L	CTTCATGTTGTCGCTTGCTAC		
P5U	TGATGAAGCTGGTCTACTTGG	SMP head protein	456
P5L	TCCATTGTGGCACGGATAGCA		
Lys-Gspa	TCGGAGCGTCAAGCCGTCTGAAAGC		
Lys-Gspb	GCAGAAAATGGATCATTGGACAAGT		
Lys-Gspc	CAAAATCGAAGTAACCATTGAGGGG		
Int-Gspa	AGCCACATCTTCCGCCATACCCTTG		
Int-Gspb	TCTAGCAGAGAACAGAGTTCCCTTG		
Int-Gspc	ACCCAAATTTACACCCATGTCACAA		

#### 1.2.2. Mitomycin C induction

To induce phage from the lysogenic strain, suspected lysogens were cultured in THB at 37°C to an optical density at 600 nm of 0.2. Mitomycin C (0.5 μg/ml) was added and incubation was continued overnight at 37°C. Induced phages were used to infect wild-type strain SS2-4 to check for plaque formation.

### 1.3. Characterization of lysogenic SS2-4

#### 1.3.1. Phenotype comparisons

Both the lysogenic and the wild-type strain SS2-4 were streaked onto THB agar and blood agar plates to examine the morphology (color and shape) of the colonies. They were also cultured in THB liquid medium and crystal violet coloration was observed under the light microscope ([Magnification: 40×], OLYMPUS, CX21FS1, Japan) **b**Growth of lysogenic and wild-type strains was compared in THB medium at 37°C during the course of 15 hours, starting at 10^6^ CFU/ml. Bacterial growth was estimated by plate counts. The experiment was repeated three times and yielded reproducible results.

### 1.4. Southern blotting and hybridization

To confirm integration of the DNA of phage SMP into the chromosome of its host, the DNA of SMP, wild SS2-4 and lysogenic SS2-4 were digested with restriction enzymes (*Pst*I and *Spe*I) and then hybridized with the whole SMP DNA probe by Southern hybridization. Southern hybridization was performed according to DIG High Prime DNA Labeling and Detection Starter Kit I (Roche, Germany, Cat. No. 11745832910).

### 1.5. Identification of phage integration sites

The attachment of the prophage is assumed occur between the putative integrase and the putative lysine genes [17]. Fragments flanking the insertion sites were amplified by using a walking kit (Seegene, Korea, Code No. K1501) with phage specific primers (Lys-Gspa, Lys-Gspb, Lys-Gspc, Int-Gspa, Int-Gspb and Int-Gspc) (Table 1). Lysogenic DNA was extracted using a DNA extraction kit (Geneaid, Taiwan, Cat. No. DF100). PCR products were loaded and analyzed by 1% agarose gel electrophoresis and the specific bands were purified using a rapid gel extraction kit (Geneaid, Taiwan, Cat. No. DF100) and sequenced (Invitrogen, Nanjing, China).

### 1.6. Sensitivity to lysozyme

Wild-type and lysogenic bacteria were cultured in THB at 37°C to an optical density at 600 nm of 0.5. This culture (1 ml) was then washed twice with one volume of 10 mM sodium phosphate buffer (pH 7.5) and the concentration of bacteria was adjusted to 1×10^8^ CFU/ml. After addition of lysozyme (Sigma, USA, Cat. No. L6876) to a final concentration of 100 ng/μl, the mixture was incubated at 37°C for 3 h. Bacterial viability was then determined by plate counting [18], [19]. Phage progeny in lysogen cultures treated with lysozyme was measured and compared to untreated controls as previously described [16]. Briefly, samples of cultures were centrifuged at 5,000×*g* for 15 min and the phage-containing supernatant was sterilized by passage through a sterilized membrane filter (0.22 μm). An aliquot of the remaining culture was plated to determine the number of PFU.

### 1.7. Virulence testing

To test the lysogenic strain for alterations in virulence relative to the wild-type, a comparative assay was carried out in separate experiments by using six groups of zebrafish for each individual strain as previously described [20], [21]. Briefly, 180 zebrafish were randomly classified into 12 groups (15 fish per group). All zebrafish were anesthetized with tricaine methane sulfonate (MS-222, Hangzhou Animal Medicine Factory) at a concentration of 95 mg/liter. Ten groups of experimental fish were injected intraperitoneally with 10 μl of either wild-type or lysogenic bacteria at five different concentrations: 1×10^8^, 1×10^7^, 1×10^6^, 1×10^5^ and 1×10^4^ CFU/fish (Table 2). Two groups of control fish were injected with 10 μl of sterile THB. Mortality was monitored for one week post-infection. The experiment was repeated three times and the lethal dose, 50% value (LD_50_) was calculated by using the method described by Reed and Muench [22].

**Table 2:** Mortality and LD_50_ of wild-type and lysogenic SS2-4.

Dose (CFU/fish)	Mortality					
	Test 1		Test 2		Test 3	
	Wild-type SS2-4	Lysogenic SS2-4	Wild-type SS2-4	Lysogenic SS2-4	Wild-typeSS2-4	Lysogenic SS2-4
10^8^	15/15	15/15	15/15	15/15	15/15	15/15
10^7^	15/15	15/15	14/15	15/15	15/15	15/15
10^6^	12/15	14/15	11/15	13/15	13/15	14/15
10^5^	6/15	8/15	6/15	9/15	5/15	8/15
10^4^	0/15	2/15	1/15	2/15	1/15	3/15
LD_50_	2.1×10^5^	6.4×10^4^	2.34×10^5^	6.92×10^4^	1.95×10^5^	6.61×10^4^

Average LD_50_ 2.13×10^5^ (wild-type SS2-4) and 6.64×10^4^ (lysogenic SS2-4).

Survival curves were made by using GraphPad Prism 5. The probability curves of survival of fish infected with wild-type or lysogenic SS2-4 at doses of 10^8^, 10^7^ and 10^6^ CFU/fish were calculated according to the Kaplan-Meier method [23].

Bacterial load was measured as previously described [24] with some modifications. Three fish from each group were sacrificed at different time-points during the infection (2, 14 and 23 h post-injection), and bacterial load in zebrafish was calculated by homogenizing fish samples in PBS. Serial dilutions of each homogenate were plated in duplicate on THB agar for enumeration of CFU.

Our experiment was conducted with the permission of the Ministry of Science and Technology of Jiangsu Province. The license number is SYXK(SU)2010-0005. All surgical procedures were performed under tricaine methane sulfonate anesthetic (MS-222, Hangzhou Animal Medicine Factory), and all efforts were made to minimize suffering.

### 1.8. DNA sequence of phage SMP

Phage DNA was isolated from the lysate by phenol-chloroform-isoamyl alcohol extraction and concentrated by ethanol precipitation as previously described [8].

Highly purified phage DNA was used for paired-end Illumina sequence analysis. The sequences were mapped against the sequence of SMP (Embank ID: EF116926).

### 1.9. Statistical analysis

Statistical analyses were performed using GraphPad Prism 5. Virulence testing, growth curve and lysozyme sensitivity experiments were carried out in triplicate. Statistical analysis was performed using Student's *t*-tests, with *P*<0.05 considered to indicate statistical significance.

## Results

### 1.1. Selection of lysogenic SS2-4

SS2-4 colonies surviving lysis by phage SMP were selected in a plate assay. To test the lysogenic characteristics of the colonies selected, colonies were checked for the presence of SMP specific genes by PCR. In all lysogens five randomly selected SMP genes were amplified by PCR (results not shown). Moreover, attempts to infect the lysogens with phage SMP were unsuccessful. Phages which could be induced from the lysogenic SS2-4 using mitomycin C were able to form plaques on the wild-type strain SS2-4. These results clearly indicate that lysogenic SS2-4 isolates have been selected. The rate of spontaneous induction of the lysogenic strain was decreased after subculture from 6.25×10^5^ PFU/ml in the first generation to 5.75×10^4^ PFU/ml in the second generation, which remained constant in later generations.

### 1.2. Characterization of lysogenic SS2-4

#### 1.2.1. Phenotype comparisons

**Figure 1:**
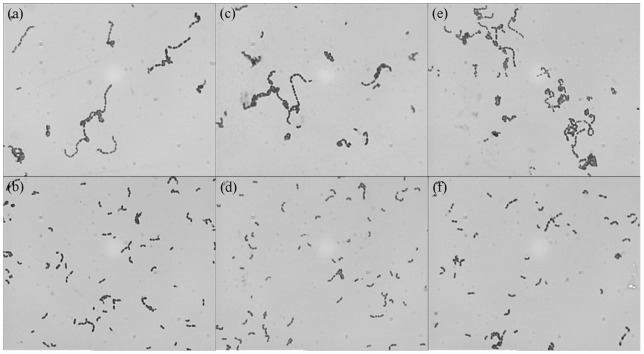
Comparison of wild-type and lysogenic SS2-4 cell morphology. (a) Wild-type SS2-4 in mid-log growth phase; (b) Lysogenic SS2-4 in mid-log growth phase; (c) Wild-type SS2-4 in the stationary growth phase; (d) Lysogenic SS2-4 in the stationary growth phase; (e) Wild-type SS2-4 in the declining growth phase; (f) Lysogenic SS2-4 in the declining growth phase. In all three phases, the lysogenic strain had a shorter chain than the wild-type strain.

Wild-type and lysogenic SS2-4 displayed similar colony morphologies on THB and blood agar plates. In contrast, compared to the wild-type strain, the lysogen had shorter chains of cells. This phenomenon was independent of growth phase used when cultured in THB in the mid-log phase (Fig. 1(a) and Fig. 1(b)), the stationary phase (Fig. 1(c) and Fig. 1(d)) and the declining phase (Fig. 1(e) and Fig. 1(f)). Thirty chains from both wild-type and lysogenic SS2-4 in different growth phases were picked at random and the cell number of the chains was calculated. The average numbers of cells in the chains of wild-type and lysogenic SS2-4 were 20±2.2 and 5±0.4 *(P*<0.0001), respectively.

#### 1.2.2. Growth curves

**Figure 2:**
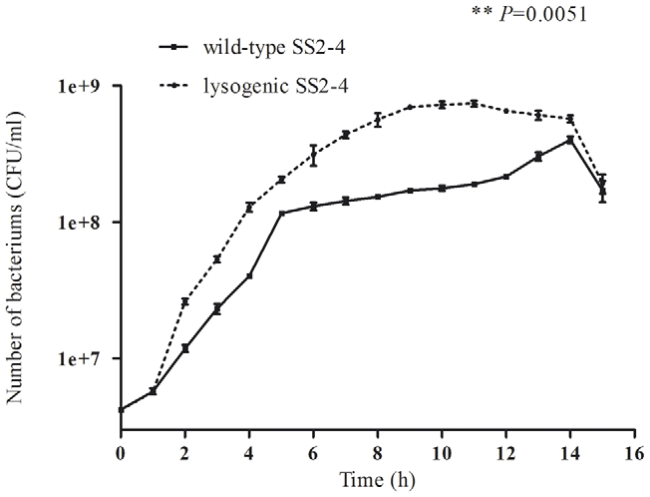
Growth curves of wild-type and lysogenic SS2-4 strains. The initial concentration of bacteria was 10^6^ CFU/ml. Number of bacteria was estimated by plate counts. The growth rate of the lysogen was more rapid than that of the wild-type and a higher plateau was maintained during the stationary phase.

Growth curves (Fig. 2) showed that the lysogenic SS2-4 grew faster and maintained a higher plateau during the stationary phase than the wild-type. A significant difference *(P* = 0.0051) was indicated in the growth rate between wild-type and lysogenic SS2-4.

### 1.3. Confirmation of phage integration into the host genome

**Figure 3:**
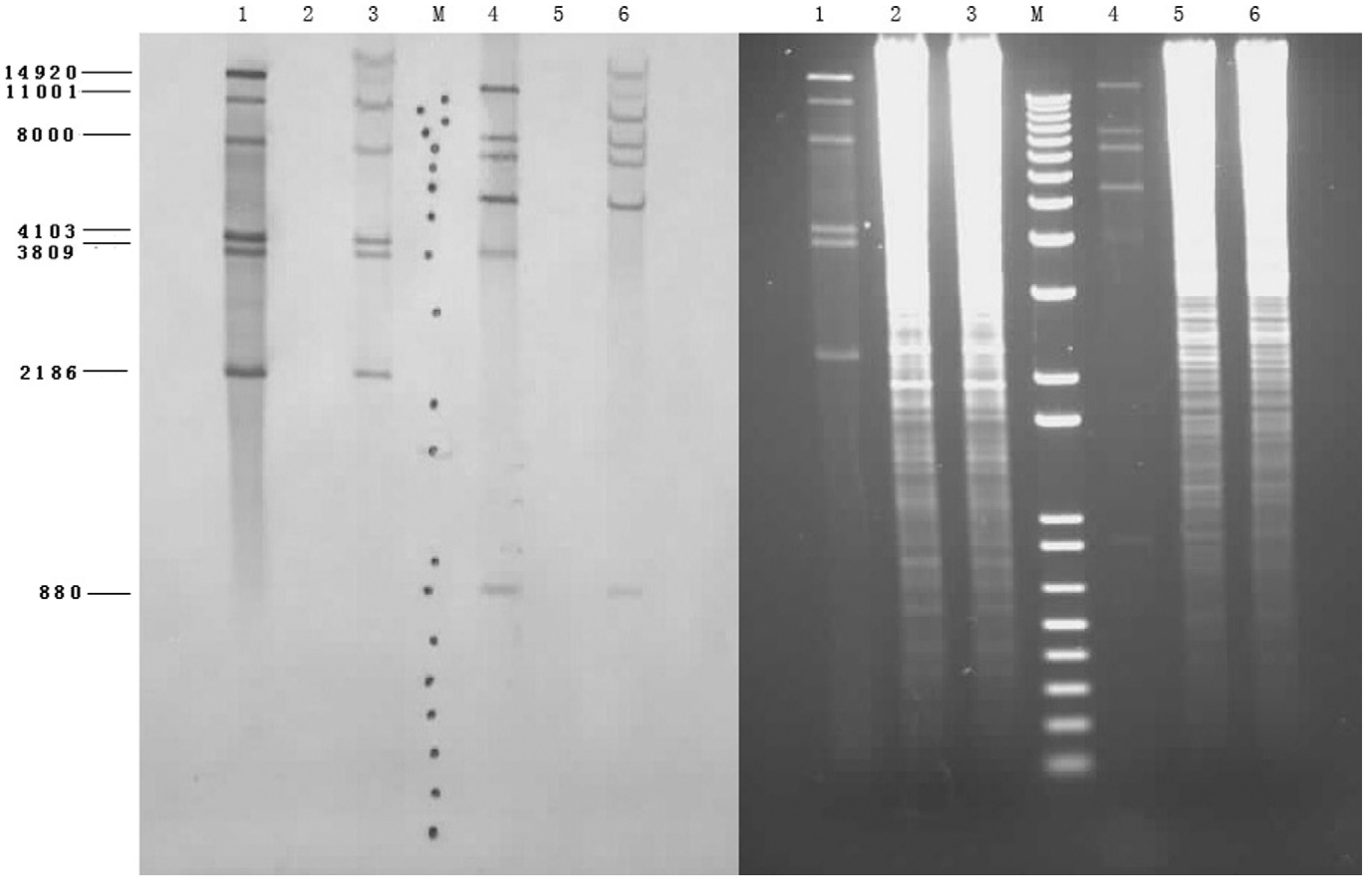
Confirmation of prophage integration by Southern hybridization. Southern gel transfer (right) and Southern hybridization (left) of SMP DNA fractions. Lanes: 1–3, SMP, wild-type SS2-4 and lysogenic SS2-4, *Pst*I digested; 4–6, SMP, wild-type SS2-4 and lysogenic SS2-4, *Spe*I digested; M, Marker.

Southern blotting and hybridization experiments were used to confirm integration the phage DNA into the chromosome of SS2-4. Therefore chromosomal DNA isolated from wild-type and lysogenic SS2-4 as well as phage SMP DNA was digested with *Pst*I and *Spe*I and hybridized with labeled SMP. The results (Fig. 3) were consistent with the insertion of the phage DNA into the chromosome of the lysogenic isolate. The 14,920 bp *Pst*I fragment as well as the 13,806 bp *Spe*I fragment present in SMP were absent in the lysogenic isolate and were replaced by two other hybridizing fragments, clearly indicating integration of the phage into the chromosome of SS2-4.

### 1.4. Identification of the phage integration sites

To determine the site of integration of the phage in the chromosome of the lysogenic isolate, fragments flanking the insertion sites were amplified by using a walking kit with phage specific primers. The choice of primers was based on the assumption that the attachment site is located between the putative integrase and the putative lysine genes. Amplified fragments obtained were subsequently sequenced. The results showed that phage SMP was inserted into the host chromosomal DNA before the DNA binding protein gene (SSU1458 in the genome of *S. suis*P1/7). We determined the attachment site to be AAAGACGCTGTAAAATAA, corresponding to bp 1,473,416 to bp 1,473,399 in the genome of *S. suis* P1/7, which does indeed lie between the integrase and lysin genes in the phage genome.

SMP has been described as a linear phage [8]. However, de novo assembly of sequences obtained for purified phage DNA clearly showed that SMP has a circular genome. The sequence of SMP in the NCBI database has been corrected (GenBank ID: EF116926.2).

### 1.5. Sensitivity to lysozyme

**Figure 4:**
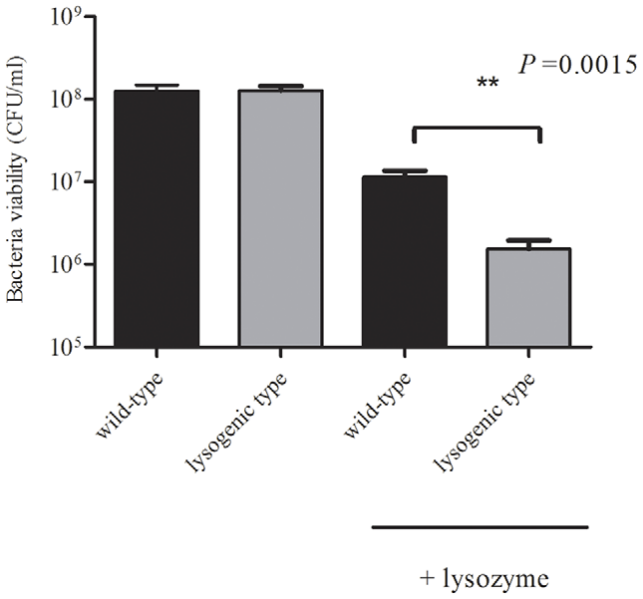
Sensitivity to lysozyme. Viable bacteria after incubation with lysozyme (3 h). First and second columns: initial bacterial viability of wild-type and lysogenic SS2-4; third and fourth columns: bacterial viability of wild-type and lysogenic SS2-4 after incubation with lysozyme (3 h).

We next evaluated the resistance of wild-type and lysogenic isolates to lysozyme. After incubation with lysozyme (3 h), the number of viable wild-type SS2-4 was reduced 12-fold (from 1.3_10^8^ CFU/ml to 1.1_10^7^ CFU/ml). In contrast, a significantly (*P* = 0.0015) greater reduction was observed following lysozyme treatment of the lysogenic isolate (86-fold reduction; from 1.3_10^8^ CFU/ml to 1.5_10^6^ CFU/ml) (Fig. 4). These data clearly indicated that the lysogenic SS2-4 is more sensitive to lysozyme than the wild-type.

To test the ability of lysozyme to induce the SMP lytic cycle, we next determined the level of phage progeny in lysogenic cultures after treatment with lysozyme. The results showed that the number of phage progeny of lysogenic strain treated with lysozyme was 2553±233 PFU/ml, while the number of phage progeny of the untreated control was 1082±90 PFU/ml.

### 1.6. Virulence testing

**Figure 5:**
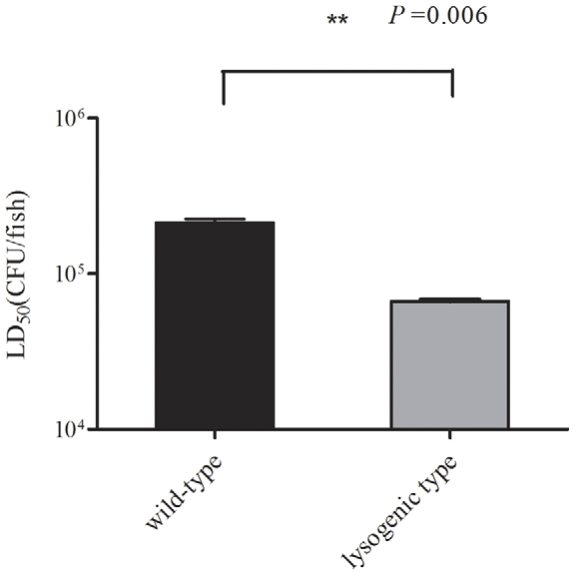
Virulence testing. Survival of wild-type and lysogenic isolates was determined in zebrafish. Left: average LD_50_ of zebrafish challenged with wild-type SS2-4; right: average LD_50_ of zebrafish challenged with lysogenic SS2-4. The Student's *t*-test was performed for comparison of the LD_50_ of the two strains using GraphPad Prism 5 software. *P* = 0.006; degrees of freedom, 2; Standard error of the mean (SEM) of LD_50_ of wild-type and lysogenic isolates was 11,360 and 1,510, respectively. A significant difference in the LD_50_ was identified between the wild-type and lysogenic SS2-4.

The mortality of the wild-type strain and lysogenic strain was evaluated in three independent experiments using the zebrafish model. Most zebrafish died within 24 h post-infection. Zebrafish injected with *S. suis* exhibited apparent signs of illness, including prolonged bleeding, loss of mobility, and dwelling at the bottom of the tank. These fish gradually lost their upright reflex and the ability to swim and finally stopped moving their gill lids. The most typical symptom observed in infected zebrafish was swelling of the abdomen. Bacteria from infected fish were isolated post-mortem and PCR analysis of selected colonies confirmed these to be *S. suis*. In contrast, the group of fish injected with THB did not demonstrate any signs of infection, nor was the presence of *S. suis* detected in internal organs. The mean LD_50_ values of the lysogenic and wild-type strains were 6.64×10^4^ CFU/fish and 2.13×10^5^ CFU/fish *(P* = 0.006), respectively (Table 2). Significant differences were identified between the LD_50_ value of wild-type and lysogenic SS2-4 (Fig. 5), indicating that phage SMP plays a role in its host pathogenicity.

**Figure 6:**
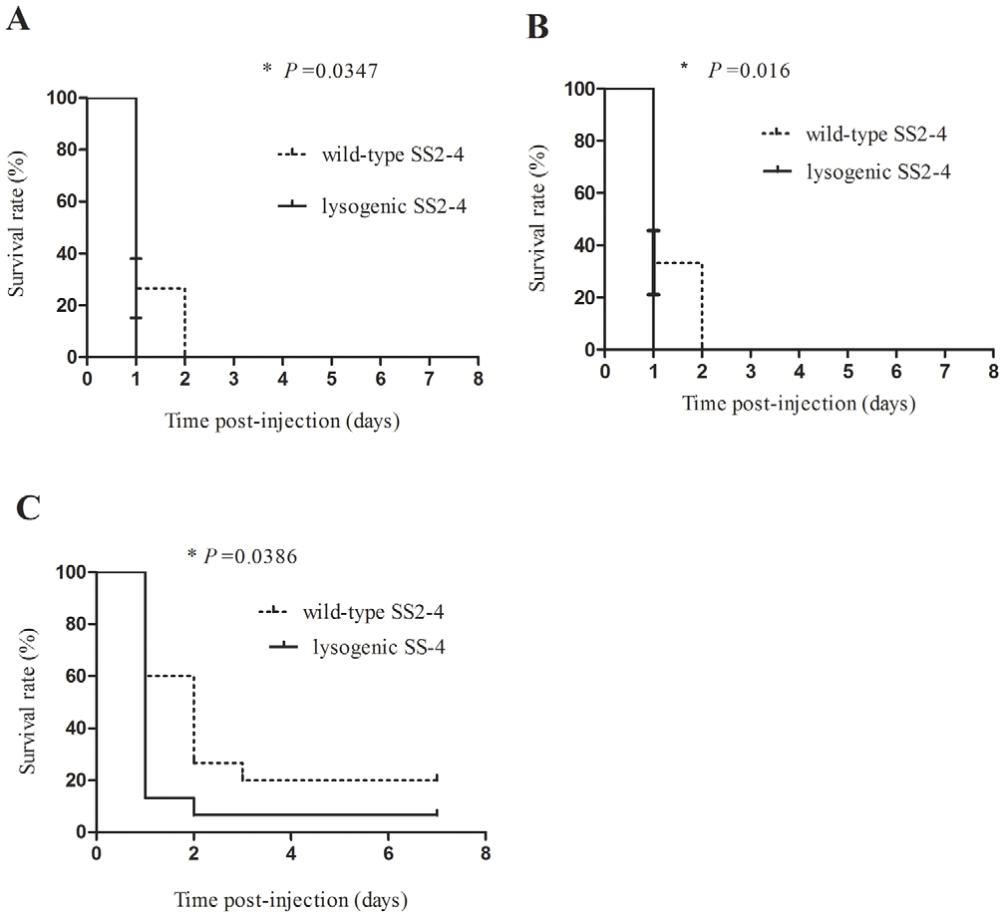
Survival curves. Survival curves of zebrafish infected with wild-type and lysogenic SS2-4 were compared using GraphPad Prism 5 software. Each experimental group contained 15 zebrafish, and each experiment was performed in triplicate. A, The survival rates of zebrafish infected with 10^8^CFU/fish of wild-type and lysogenic SS2-4; B, The survival rates of zebrafish infected with 10^7^CFU/fish of wild-type and lysogenic SS2-4; C, The survival rates of zebrafish infected with 10^6^CFU/fish of wild-type and lysogenic SS2-4.

The survival rates of infected fish were also determined to evaluate the virulence of *S. suis* using the zebrafish model of infection. Survival curves of wild-type and lysogenic SS2-4 were compared at each dose. Significant differences in the survival curves of fish infected with doses of 10^8^, 10^7^ and 10^6^ CFU/ fish were identified between the wild-type and lysogenic SS2-4 (*P* = 0.0347, 0.016, 0.0386, respectively [Fig. 6A, Fig. 6B and Fig. 6C]).

**Figure 7:**
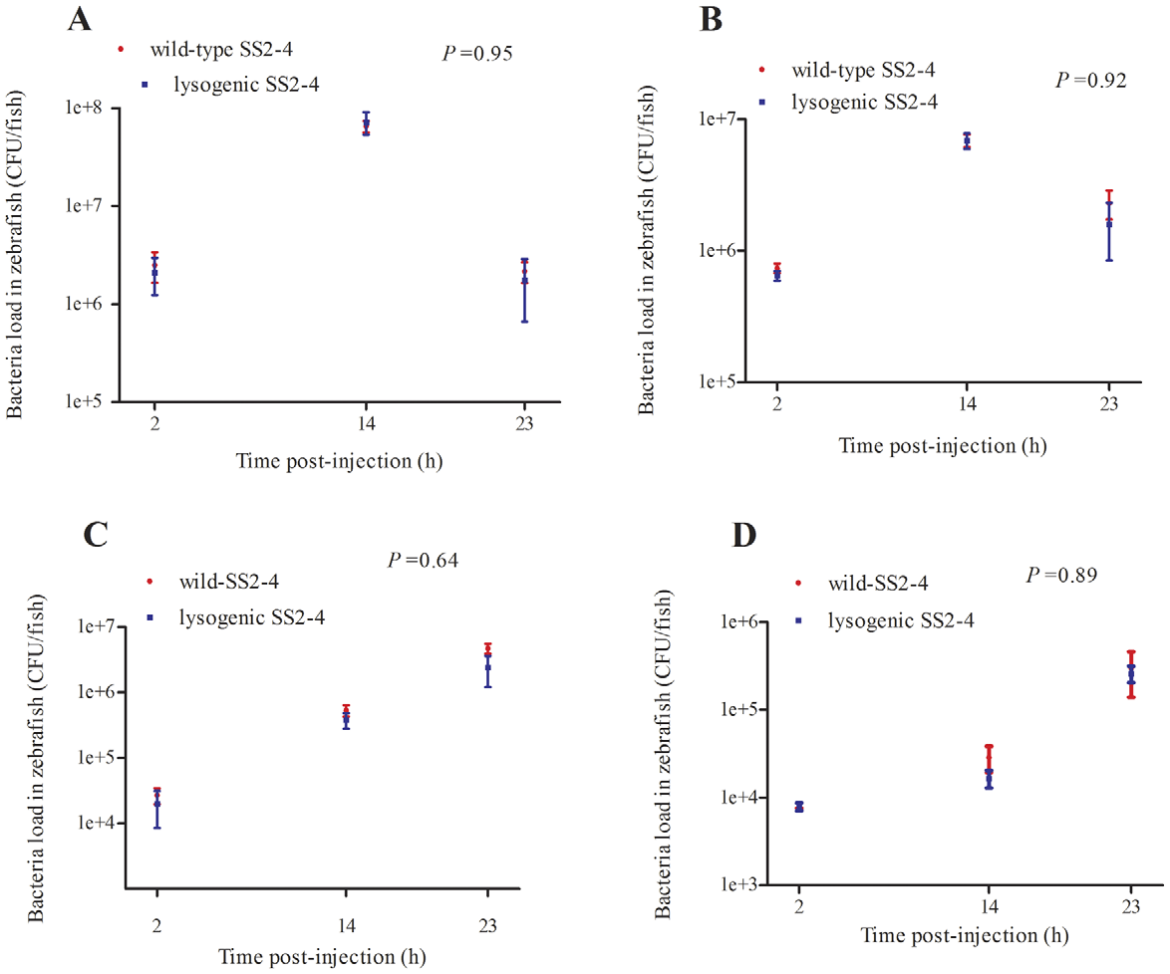
Bacterial load of zebrafish infected with wild-type or lysogenic SS2-4. Three infected fish from each dose group were sacrificed at 2, 14, and 23 h post-infection. A, B, C, D: the bacterial load of fish infected with wild-type and lysogenic SS2-4 at 10^8^ CFU/fish, 10^7^ CFU/fish, 10^6^ CFU/fish, 10^5^ CFU/fish, respectively. The Student's *t*-test was performed for comparison of the bacterial load at each dose between wild-type and lysogenic SS2-4 using GraphPad Prism 5 software.

The bacterial load of each group was measured at different time-points during the infection (2, 14 and 23 h post-injection). No significant difference was observed in the growth rate in vivo between the wild-type and lysogenic SS2-4. However, the bacterial load of fish infected with wild-type SS2-4 was slightly higher than that of the lysogenic SS2-4 in vivo (Fig. 7A, Fig. 7B, Fig. 7C and Fig. 7D).

## Discussion

Zebrafish were infected with wild-type or lysogenic SS2-4 by intraperitoneal injection. LD_50_ and survival analyses were performed to evaluate the pathogenesis of the two strains. The average LD_50_ of the two strains differed significantly (*P* = 0.006). Comparison of the survival curves also showed significant difference between the two strains at doses of 10^8^, 10^7^ and 10^6^ CFU/fish. There was no significant difference in the survival rates of infected fish between the wild-type and lysogenic SS2-4 at doses of 10^5^ and 10^4^ CFU/fish due to low virulence of these low doses in fish. To exclude the possibility that the observed increase in virulence was merely an effect of the increased growth rate of the lysogenic isolate, the bacterial load of the infected fish in each group was evaluated at different time-points (2, 14 and 23 h) post-infection. The bacterial load of the two strains did not differ significantly and was independent of the infection dose used. These results indicate that addition of prophage led to a significant increase in the virulence of this strain. This study is the first attempt to lysogenize a *S. suis* strain in vitro with a known bacteriophage in order to study the role of the phage in pathogenesis. In several species, such as *Salmonella*, *Staphylococcus aureus*, *Enterococcus faecalis* and *Streptococcus pneumonia* prophages have been shown to play a role in the virulence of the lysogen and to contain putative VFs [25], [26], [27], [28]. Phage acquired genes may encode secreted VFs as well as cell wall envelope factors that aid in the establishment of animal infections [25]. Here we showed that SMP as a prophage contributed to *S. suis* pathogenicity. The SMP was shown to integrate into the upstream of the DNA binding protein gene (SSU1458 in the genome of *S. suis* P1/7) without host gene disruption. Furthermore, no known virulence genes carried by SMP were identified [29]. However, contribution of the prophage SMP to *S. suis* pathogenicity was demonstrated. It can be speculated that this is because phage SMP may encode a novel virulence factor or the prophage is involved in the regulation of the host. Furthermore, that the prophage is against lysogenic host by at least two processes: the metabolic burden of replication of additional DNA and the lysis of the host after prophage induction. To compensate for these disadvantages, it can be hypothesized that temperate phages encode functions that increase the fitness of the lysogen [30].Here, the prophage SMP might invoke some mechanism to increase the fitness of the lysogen, such as increase in virulence and a more rapid growth rate. In coliphage λ, phage encoded repressors have been shown to modulate host metabolic gene expression and phenotype, resulting in economization of host energy expenditure [31]. Comparison of lysogenized marine bacteria to the uninfected hosts indicated that prophage acquisition is correlated with host metabolic gene suppression [31]. The prophage SMP also has the repressor gene and it might have similar function to the coliphage λ repressor.

The lysogenic strain is much more sensitive to lysozyme than the wild-type. The bacteriophage life cycle involves two multiplication processes: the lytic cycle and lysogenic cycle. When the prophage is “triggered”, the phage genome detaches from the host cell DNA and enters the lytic cycle. While it is currently unclear what exactly constitutes a “trigger” that activates the phage DNA from the latent stage into the in lytic cycle, prophage induction is usually caused by cellular damage [32], [33]. Here, lysozyme might be a “trigger”, which caused disruption of the cell wall followed by release of the daughter progeny into the environment. Indeed, the number of phage progeny in lysogenic cultures treated with lysozyme was two-fold higher than that in the untreated control, thus implicating lysozyme as a “trigger” of liberation and provision of additional resources to the lysogens in the form of liberated nutrients.

The lysogenic strain displayed decreased chain length compared to the wild-type strain. Regulation of chain length by chain-forming bacteria is believed to depend on wall-associated autolytic activity and environmental conditions [34], [35]. The rate of spontaneous induction of the lysogenic strain was maintained at a high level (6.1×10^4^ PFU/ml) after subculture. The shorter chains of the lysogenic strain may be caused by disruption of the cell wall by spontaneously induced prophages. The rate of spontaneous induction of the lysogenic strain in the first generation was much higher than in the subculture, which might due to instability of the first generation.

Another physiological change was indicated by the more rapid growth rate of the lysogenic SS2-4 strain compared with the wild-type. This was consistent with reports on *Streptococcus pneumonia* isolates containing MM1 phage in which the lysogenic strain 8 grew faster than the cured strain 8C [27]. Some classical data also indicated that *E. coli* cells containing prophages (lambda, Mu, P2, and even cryptic prophages) grow more rapidly than non-lysogenic *E. coli* strains [36], [37], [38]. This might be related to gene expression of prophages [9].

Infection of zebrafish has been previously demonstrated using *S. suis* [20], [39], [40], [41]. Moreover, zebrafish as infection model for *S. suis* has been accepted by Office International Des Epizooties (OIE) (http://web.oie.int/eng/info/hebdo/AIS_54.HTM). Therefore, in this study, the virulence of these two SS2 strains was evaluated in zebrafish in order to demonstrate that these SS2 strains exhibit differences in pathogenesis.

## Conclusions

The present study is the first attempt to lysogenize a *S. suis* strain in vitro with a known bacteriophage in order to study the role of the phage in pathogenesis. Several *S. suis* strains harboring prophages have been detected in our laboratory (data not shown), although their genetic contents have not yet been well characterized. This study will expand the observations on the evolution and epidemiology of Streptococcal infections.

## References

[1] Staats JJ, Feder I, Okwumabua O, Chengappa MM (1997) Streptococcus suis: past and present. Vet Res Commun 21: 381–407. 9266659 10.1023/a:1005870317757

[2] Trottier S, Higgins R, Brochu G, Gottschalk M (1991) A case of human endocarditis due to Streptococcus suis in North America. Rev Infect Dis 13: 1251–1252. 1775866 10.1093/clinids/13.6.1251

[3] Nga TV, Nghia HD, Tu le TP, Diep TS, Mai NT, et al (2011) Real-time PCR for detection of Streptococcus suis serotype 2 in cerebrospinal fluid of human patients with meningitis. Diagn Microbiol Infect Dis 70: 461–467. 21767702 10.1016/j.diagmicrobio.2010.12.015 PMC3146703

[4] Hu X, Zhu F, Wang H, Chen S, Wang G, et al (2000) [Studies on human streptococcal infectious syndrome caused by infected pigs]. Zhonghua Yu Fang Yi Xue Za Zhi 34: 150–152. 11860922

[5] Harel J, Martinez G, Nassar A, Dezfulian H, Labrie SJ, et al (2003) Identification of an inducible bacteriophage in a virulent strain of Streptococcus suis serotype 2. Infect Immun 71: 6104–6108. 14500539 10.1128/IAI.71.10.6104-6108.2003 PMC201037

[6] Hu P, Yang M, Zhang A, Wu J, Chen B, et al (2011) Complete genome sequence of Streptococcus suis serotype 14 strain JS14. J Bacteriol 193: 2375–2376. 21398551 10.1128/JB.00083-11 PMC3133069

[7] Hu P, Yang M, Zhang A, Wu J, Chen B, et al (2011) Complete genome sequence of Streptococcus suis serotype 3 strain ST3. J Bacteriol 193: 3428–3429. 21572001 10.1128/JB.05018-11 PMC3133292

[8] Ma YL, Lu CP (2008) Isolation and identification of a bacteriophage capable of infecting Streptococcus suis type 2 strains. Vet Microbiol 132: 340–347. 18676101 10.1016/j.vetmic.2008.05.013

[9] Brussow H, Canchaya C, Hardt WD (2004) Phages and the evolution of bacterial pathogens: from genomic rearrangements to lysogenic conversion. Microbiol Mol Biol Rev 68: 560–602. 15353570 10.1128/MMBR.68.3.560-602.2004 PMC515249

[10] Beutin L, Kaulfuss S, Cheasty T, Brandenburg B, Zimmermann S, et al (2002) Characteristics and association with disease of two major subclones of Shiga toxin (Verocytotoxin)-producing strains of Escherichia coli (STEC) O157 that are present among isolates from patients in Germany. Diagn Microbiol Infect Dis 44: 337–346. 12543538 10.1016/s0732-8893(02)00474-1

[11] Carrolo M, Frias MJ, Pinto FR, Melo-Cristino J, Ramirez M (2010) Prophage spontaneous activation promotes DNA release enhancing biofilm formation in Streptococcus pneumoniae. PLoS One 5: e15678. 21187931 10.1371/journal.pone.0015678 PMC3004956

[12] Nagiec MJ, Lei B, Parker SK, Vasil ML, Matsumoto M, et al (2004) Analysis of a novel prophage-encoded group A Streptococcus extracellular phospholipase A(2). J Biol Chem 279: 45909–45918. 15304506 10.1074/jbc.M405434200

[13] Dmitriev AV, McDowell EJ, Kappeler KV, Chaussee MA, Rieck LD, et al (2006) The Rgg regulator of Streptococcus pyogenes influences utilization of nonglucose carbohydrates, prophage induction, and expression of the NAD-glycohydrolase virulence operon. J Bacteriol 188: 7230–7241. 17015662 10.1128/JB.00877-06 PMC1636216

[14] Lindsay AM, Zhang M, Mitchell Z, Holden MT, Waller AS, et al (2009) The Streptococcus equi prophage-encoded protein SEQ2045 is a hyaluronan-specific hyaluronate lyase that is produced during equine infection. Microbiology 155: 443–449. 19202092 10.1099/mic.0.020826-0

[15] Pasharawipas T, Thaikua S, Sriurairatana S, Ruangpan L, Direkbusarakum S, et al (2005) Partial characterization of a novel bacteriophage of Vibrio harveyi isolated from shrimp culture ponds in Thailand. Virus Res 114: 63–69. 16023243 10.1016/j.virusres.2005.05.012

[16] Shkilnyj P, Koudelka GB (2007) Effect of salt shock on stability of lambdaimm434 lysogens. J Bacteriol 189: 3115–3123. 17307857 10.1128/JB.01857-06 PMC1855845

[17] Groth AC, Calos MP (2004) Phage integrases: biology and applications. J Mol Biol 335: 667–678. 14687564 10.1016/j.jmb.2003.09.082

[18] Metcalf RH, Deibel RH (1973) Effect of lysozyme on enterococcal viability in low ionic environments. J Bacteriol 113: 278–286. 4569405 10.1128/jb.113.1.278-286.1973 PMC251628

[19] Shimada J, Moon SK, Lee HY, Takeshita T, Pan H, et al (2008) Lysozyme M deficiency leads to an increased susceptibility to Streptococcus pneumoniae-induced otitis media. BMC Infect Dis 8: 134. 18842154 10.1186/1471-2334-8-134 PMC2575207

[20] Wu Z, Zhang W, Lu C (2008) Comparative proteome analysis of secreted proteins of Streptococcus suis serotype 9 isolates from diseased and healthy pigs. Microb Pathog 45: 159–166. 18554861 10.1016/j.micpath.2008.04.009

[21] Pu JunYi HX, Lu ChengPing (2007) Virulence detection of Streptococcus suis type 2 in zebrafish. Scientia Agricultura Sinica. pp.2655–2658.

[22] Reed LJ, Muench H (1938) A simple method of estimating fifty percent endpoints. The American Journal of Hygiene 27: 493–497.

[23] Lin H, Wei JC, Tan CY, Liu YY, Li YH, et al.. (2012) Survival analysis of late-onset systemic lupus erythematosus: a cohort study in China. Clin Rheumatol. 10.1007/s10067-012-2073-6 22983769

[24] Buchanan JT, Stannard JA, Lauth X, Ostland VE, Powell HC, et al (2005) Streptococcus iniae phosphoglucomutase is a virulence factor and a target for vaccine development. Infect Immun 73: 6935–6944. 16177373 10.1128/IAI.73.10.6935-6944.2005 PMC1230984

[25] Bae T, Baba T, Hiramatsu K, Schneewind O (2006) Prophages of Staphylococcus aureus Newman and their contribution to virulence. Mol Microbiol 62: 1035–1047. 17078814 10.1111/j.1365-2958.2006.05441.x

[26] Figueroa-Bossi N, Bossi L (1999) Inducible prophages contribute to Salmonella virulence in mice. Mol Microbiol 33: 167–176. 10411733 10.1046/j.1365-2958.1999.01461.x

[27] Loeffler JM, Fischetti VA (2006) Lysogeny of Streptococcus pneumoniae with MM1 phage: improved adherence and other phenotypic changes. Infect Immun 74: 4486–4495. 16861634 10.1128/IAI.00020-06 PMC1539626

[28] Yasmin A, Kenny JG, Shankar J, Darby AC, Hall N, et al (2010) Comparative genomics and transduction potential of Enterococcus faecalis temperate bacteriophages. J Bacteriol 192: 1122–1130. 20008075 10.1128/JB.01293-09 PMC2812964

[29] Fittipaldi N, Segura M, Grenier D, Gottschalk M (2012) Virulence factors involved in the pathogenesis of the infection caused by the swine pathogen and zoonotic agent Streptococcus suis. Future Microbiol 7: 259–279. 22324994 10.2217/fmb.11.149

[30] Canchaya C, Proux C, Fournous G, Bruttin A, Brussow H (2003) Prophage genomics. Microbiol Mol Biol Rev 67: 238–276, table of contents. 10.1128/MMBR.67.2.238-276.2003 PMC156470 12794192

[31] Paul JH (2008) Prophages in marine bacteria: dangerous molecular time bombs or the key to survival in the seas? ISME J 2: 579–589. 18521076 10.1038/ismej.2008.35

[32] Marsh P, Wellington EMH (1994) phage-host interactions in soil. FEMS Microbiology Ecology 15: 99–108.

[33] Rozanov DV, D'Ari R, Sineoky SP (1998) RecA-independent pathways of lambdoid prophage induction in Escherichia coli. J Bacteriol 180: 6306–6315. 9829941 10.1128/jb.180.23.6306-6315.1998 PMC107717

[34] Thibodeau EA, Ford CM (1991) Chain formation and de-chaining in Streptococcus sobrinus SL-1. Oral Microbiol Immunol 6: 313–315. 1820572 10.1111/j.1399-302x.1991.tb00500.x

[35] Rehm HJ GR, editor (2010) Biotechnology Set. New York: Wiley-VCH.

[36] Wang X, Kim Y, Ma Q, Hong SH, Pokusaeva K, et al (2010) Cryptic prophages help bacteria cope with adverse environments. Nat Commun 1: 147. 21266997 10.1038/ncomms1146 PMC3105296

[37] Edlin G, Lin L, Bitner R (1977) Reproductive fitness of P1, P2, and Mu lysogens of Escherichia coli. J Virol 21: 560–564. 319256 10.1128/jvi.21.2.560-564.1977 PMC353858

[38] Lin L, Bitner R, Edlin G (1977) Increased reproductive fitness of Escherichia coli lambda lysogens. J Virol 21: 554–559. 319255 10.1128/jvi.21.2.554-559.1977 PMC353857

[39] Pu JY, Huang XX, Lu CP (2007) Virulence Detection of Streptococcus suis Type 2 in Zebrafish. Scientia Agricultura Sinica 40: 2655–2658.

[40] Ju CX, Gu HW, Lu CP (2012) Characterization and functional analysis of atl, a novel gene encoding autolysin in Streptococcus suis. J Bacteriol 194: 1464–1473. 22228730 10.1128/JB.06231-11 PMC3294825

[41] Wang Y, Zhang W, Wu Z, Zhu X, Lu C (2011) Functional analysis of luxS in Streptococcus suis reveals a key role in biofilm formation and virulence. Vet Microbiol 152: 151–160. 21621932 10.1016/j.vetmic.2011.04.029

